# Variant allele frequency as a predictor of treatment response to osimertinib in EGFR-mutated NSCLC

**DOI:** 10.3389/fmolb.2026.1822285

**Published:** 2026-04-24

**Authors:** Walid Shalata, Bilal Krayim, Asmah Miari, Abed Agbarya, Nir Peled, Yulia Dudnik, Ahron Yehonatan Cohen, Amichay Meirovitz, Natalie Maimon Rabinovich, Alexander Yakobson, Firas Abu Akar, Ronen Brenner

**Affiliations:** 1 The Legacy Heritage Cancer Center and Dr. Larry Norton Institute, Soroka Medical Center, Beersheba, Israel; 2 Ben-Gurion University of the Negev, Beersheba, Israel; 3 Helmsley Cancer Center, Shaare Zedek Medical Center, Jerusalem, Israel; 4 Department of Oncology, Meir Medical Center, Kfar Saba, Israel; 5 Oncology Department, Bnai Zion Medical Center, Haifa, Israel; 6 Edith Wolfson Medical Center, Oncology Institute, Holon, Israel; 7 Faculty of Medicine, Tel Aviv University, Tel Aviv, Israel

**Keywords:** EGFR, non-small cell lung cancer, osimertinib, overall survival, progression-free survival, variant allele frequency

## Abstract

**Background:**

Osimertinib has been approved as a treatment option for epidermal growth factor receptor (EGFR) mutated non-small cell lung cancer (NSCLC). However, despite the identification of common mutations, there are still no additional pathological factors that can be used to predict treatment response and improvement in survival.

**Methods:**

This retrospective study utilized data from a multi-center registry of NSCLC patients with EGFR mutations who were treated with first-line osimertinib therapy between March 2017 and December 2024. The variant allele frequency (VAF) was evaluated as a potential predictive factor for overall survival (OS).

**Results:**

For the cohort of 147 patients who met eligibility requirements, the mean OS was 25.5 months (95% CI 20.28–30.75) and progression-free survival (PFS) was 21.4 months (95% CI 17.38–25.37). The mean OS was 19.7 months (95% CI 14.21–25.10) for VAF <30% and 31.4 months (95% CI 23.15–39.56) for VAF ≥30%. The mean PFS was 18.2 months (95% CI 12.94–23.46) for VAF <30% and 25.0 months (95% CI 19.14–30.94) for VAF ≥30%. In the subgroup of EGFR ex 19del the mean OS was 21.1 months (95% CI 11.04–31.25) for VAF <30% and 36.6 months (95% CI 25.61–47.50) for VAF ≥30% and for the subgroup with EGFR ex21 L858R the mean OS was 18.7 months (95% CI 12.36–25.00) for VAF <30% and 25.5 months (95% CI 13.78–37.22) for VAF ≥30%.

**Conclusion:**

Our study demonstrated that the VAF is a significant factor in predicting OS and PFS in EGFR mutant patients treated with osimertinib. Patients with a VAF ≥30% showed significantly improved OS and PFS compared to those with a VAF <30%. Specifically, the mean OS was longer for patients with higher VAF in both the overall cohort and in subgroups with EGFR exon 19 deletions and exon 21 L858R mutations. These findings suggest that VAF could serve as a valuable predictive biomarker for treatment outcomes in EGFR-mutated NSCLC patients, highlighting its potential role in personalizing treatment strategies.

## 1 Introduction

Lung cancer is the leading cause of cancer-related mortality and the second most prevalent cancer worldwide, according to recent reports. It is estimated to account for approximately 12% of new cancer cases, following prostate cancer (26%) in men and breast cancer (30%) in women. Despite this, lung cancer remains the primary cause of cancer-related deaths, responsible for nearly 45% of all cancer fatalities across both sexes. Non-small cell lung cancer (NSCLC), which includes adenocarcinoma and squamous cell carcinoma, is the most common histological type, representing around 85% of all lung cancer cases (Sung et al.; Shalata et al., 2024; Shalata et al., 2021).

Somatic activating mutations in the tyrosine kinase domain of the epidermal growth factor receptor (EGFR) occur in approximately 15% of white and nearly 50% of Asian patients with advanced NSCLC. EGFR tyrosine kinase inhibitors (EGFR-TKIs) act as competitive inhibitors, binding to the adenosine triphosphate (ATP) binding sites on EGFR. This prevents EGFR autophosphorylation and the activation of downstream signaling pathways, effectively inhibiting tumor cell proliferation and metastasis. As a result, EGFR-TKIs play a pivotal role in the targeted treatment of NSCLC. The use of EGFR-TKIs has notably improved the prognosis for patients with EGFR-sensitive mutations in NSCLC, with modest improvements observed in progression-free survival (PFS), overall response rate (ORR), and OS (Zhang et al., 2016; Tan et al., 2015; Tan et al., 2018; Cross et al., 2014).

Currently, three generations of EGFR-TKIs have been approved for treating NSCLC patients with EGFR mutations in various clinical scenarios. First-generation EGFR-TKIs (gefitinib, erlotinib, and icotinib) and second-generation EGFR-TKIs (afatinib and dacomitinib) have demonstrated substantial clinical benefit for patients with EGFR exon 19 deletions and EGFR exon 21 p.L858R mutations in advanced NSCLC (Soria et al., 2018; Lamb, 2021).

Osimertinib, an irreversible third-generation EGFR-TKI, targets both EGFR-sensitizing mutations and the T790M resistance mutation. It binds covalently to the EGFR tyrosine kinase domain, specifically targeting the cysteine residue at position 797 within the ATP-binding site. Osimertinib is recommended as the first-line treatment for adults with metastatic EGFR-mutated NSCLC. This recommendation is based on the FLAURA trial, a double-blind, phase three study that compared osimertinib to first-generation EGFR-TKIs (gefitinib and erlotinib) in patients with EGFR mutation-positive NSCLC (Soria et al., 2018; Lamb, 2021; Meng et al., 2023; D'Angelo et al., 2011).

Variant allele frequency (VAF) refers to the proportion of sequencing reads that contain a particular genetic variant within a given locus, providing valuable information about tumor heterogeneity. It can also help distinguish between driver and passenger mutations, as well as indicate whether certain genomic alterations are likely germline in origin (Fri et al., 2021; Gieszer et al., 2021). While accurately determining the clonal proportion can be difficult computationally, the allelic frequency is easily accessible and could serve as a useful surrogate marker. The broader impact of VAF on osimertinib response, however, is not yet fully understood, highlighting the need for further exploration. In this study, we present real-world data examining the association between VAF and the efficacy of osimertinib, including its effects on OS and progression-free survival (PFS).

## 2 Materials and methods

### 2.1 Patient selection

This retrospective, non-interventional observational study was conducted across multiple institutions, focusing on patients diagnosed with NSCLC and EGFR mutations who received osimertinib as their first-line treatment between March 2017 and December 2024. Data collection continued until December 2024. The collected information encompassed patient details such as age at diagnosis, sex, treatment regimen, OS, PFS, therapy start and end dates, smoking history, sites of metastasis, PD-L1 status, the specific type of EGFR mutation (EGFR exon 19 deletions and EGFR exon 21 p.L858R), the date of the last follow-up, progression sites, Eastern Cooperative Oncology Group (ECOG) performance status scores and any recorded toxicities. All patients underwent evaluation by a multidisciplinary team, which included medical oncologists, pulmonologists, radiation oncologists, pathologists, imaging and nuclear physicians, and thoracic surgeons. These evaluations were based on pathological findings, imaging results, and the patient’s performance status. Each patient was assigned a primary physician responsible for managing their treatment. For patients diagnosed with advanced or metastatic disease (any T, N 1–3, and/or M1), medical oncologists took the lead in their care, following the guidelines set by the National Comprehensive Cancer Network (NCCN) (National Comprehensive Cancer Network).

### 2.2 Patient monitoring, clinical information, and data collection

Prior to initiating treatment, patients underwent disease staging using brain magnetic resonance imaging (MRI), total-body computed tomography (CT) scans, or fluorodeoxyglucose (FDG) positron emission tomography-computed tomography (PET-CT). Measurable target lesions, including those with NSCLC EGFR mutations identified through follow-up or imaging, were assessed. Treatment responses were evaluated by the oncologists using the Response Evaluation Criteria in Solid Tumors (RECIST). Safety monitoring was conducted using the Common Terminology Criteria for Adverse Events (CTCAE), version 5.0. During the treatment period, radiologic re-evaluations, such as MRI of the brain, CT, or FDG-PET-CT, were performed every three to 4 months.

Tumor samples underwent molecular analysis as part of routine clinical evaluation in the participating pathology laboratories. VAF was primarily determined using Next-Generation Sequencing (NGS) platforms following the implementation of molecular profiling in the pathology workflow. In selected cases, detected variants were subsequently verified using polymerase chain reaction (PCR)-based methods to confirm the presence of mutations and ensure analytical validity. All molecular analyses were performed in certified pathology laboratories according to standard clinical protocols to ensure reproducibility and reliability of VAF measurements.

### 2.3 Inclusion criteria

Patients were eligible for the study if they met several criteria. They had to be 18 years or older and diagnosed with histologically confirmed NSCLC with EGFR mutations, specifically in exon 21 or exon 19, which were validated through tissue biopsy. Additionally, patients needed to have known values of VAF and must have received osimertinib as their sole first-line treatment.

### 2.4 Exclusion criteria

Patients were excluded from the study if they had received any systemic cancer therapy in the 4 years prior to the study or if their VAF values were unclear. This exclusion criterion was put in place to ensure that the study results were not impacted by previous treatments and patients who received osimertinib as a later-line treatment.

## 3 Results

This was a retrospective, non-interventional, multicentre real-world study analyzing data from 147 patients with locally advanced or metastatic NSCLC, identified with either EGFR exon 19 deletions or EGFR exon 21 L858R mutations. These patients were treated between March 2017 and December 2024. Their demographic, clinical, and pathological characteristics, as well as mutation subtypes and associated co-mutations were recorded Our patients had a mean age of 71.3 years (range 25–90); females had a mean age of 71.1 years, while males had a slightly higher mean age of 71.8 years. The majority of participants were female (74.83%), and most were diagnosed as Stage 4 (85.71%). Most participants had a good ECOG performance status at diagnosis, with 82.31% having an ECOG score of 0 or 1. In terms of smoking history, 27.21% were former or current smokers at diagnosis, while 72.79% had never smoked. All participants had lung adenocarcinoma with EGFR mutations, with 54.42% having EGFR exon 19 deletions and 45.58% carrying the EGFR exon 21 L858R mutation. The mean VAF for exon 19 deletions was 36.65%, while for exon 21 L858R it was 33.9%. Regarding metastasis at diagnosis, lung metastasis was most common (82.31%), followed by pleural (36.73%), bone (35.37%), and brain (30.61%) metastases. Fewer participants had liver (15.65%) or adrenal (5.44%) metastasis. Lymph node involvement was noted in 49.66% of participants, are presented in Table 1.

**TABLE 1:** Baseline characteristics of the study population (n = 147).

Characteristics		Mean (range)
Age (years)		71.3 (25–90)
	Female	71.1 (38–90)
	Male	71.8 (25–90)
Gender		Frequencies (percentage)
	Female	110 (74.83)
	Male	37 (25.17)
ECOG status (at diagnosis)		
	0	52 (35.37)
	1	69 (46.94)
	2	18 (12.24)
	3	8 (5.44)
Stage at diagnosis		
	3	21 (14.29)
	4	126 (85.71)
Smoking status (at diagnosis)		
	Former or current	40 (27.21)
	Never	107 (72.79)
Histology	Adenocarcinoma	147 (100)
Type of histology	Tissue	147 (100)
EGFR mutation types		Mean (median)
	EGFR ex 19 deletions	80 (54.42)
		Mean (range)
	VAF	36.65 (2.2–96.16)
		Mean (median)
	EGFR ex 21 L858R	67 (45.58)
		Mean (range)
	VAF	33.9 (2.7–94.54)
Metastasis sites at diagnosis		
Lung		
	Yes	121 (82.31)
	No	26 (17.69)
Pleural		
	Yes	54 (36.73)
	No	93 (63.27)
Bone		
	Yes	52 (35.37)
	No	95 (64.63)
Brain		
	Yes	45 (30.61)
	No	102 (69.39)
Liver		
	Yes	23 (15.65)
	No	124 (84.35)
Adrenal		
	Yes	8 (5.44)
	No	139 (94.56)
Lymph nodes		
	Yes	73 (49.66)
	No	74 (50.34)

**FIGURE 1:**
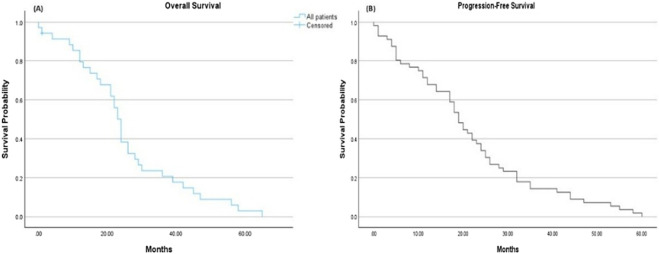
Kaplan-Meier survival estimates. **(A)** Overall survival: The mean overall survival was 25.5 months (95% CI 20.28–30.75). **(B)** Progression-free survival: The mean progression-free survival was 21.4 months (95% CI 17.38–25.37).

Regarding the efficacy of osimertinib, the mean OS for the overall population was 25.5 months (95% CI: 20.28–30.75), while the mean PFS was 21.4 months (95% CI: 17.38–25.37) (Figures 1A,B).

**FIGURE 2:**
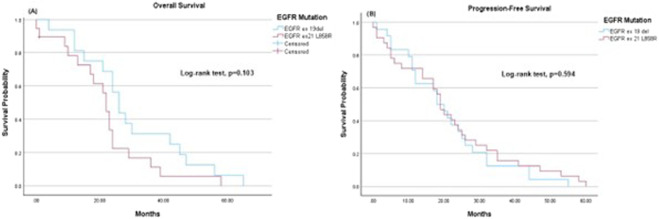
Kaplan-Meier survival estimates. **(A)** Overall survival: The mean overall survival was 29.8 months (95% CI 21.52–38.11) for EGFR ex 19del and 21.8 months (95% CI 15.48–28.02) for EGFR ex 21 L858R. **(B)** Progression-free survival: The mean progression-free survival was 20.7 months (95% CI 15.26–26.16) for EGFR ex 19del and 21.9 months (95% CI 16.13–27.62) for EGFR ex 21 L858R.

Subgroup analyses revealed differences in survival outcomes based on EGFR mutation subtypes. Patients with EGFR exon 19 deletion (ex19del) had a mean OS of 29.8 months (95% CI: 21.52–38.11), compared to 21.8 months (95% CI: 15.48–28.02) for those with EGFR exon 21 L858R. However, the difference was not statistically significant (Log-rank test, p = 0.103). Similarly, the mean PFS was 20.7 months (95% CI: 15.26–26.16) for EGFR ex19del and 21.9 months (95% CI: 16.13–27.62) for EGFR exon 21 L858R (Log-rank test, p = 0.594) (Figures 2A,B).

**FIGURE 3:**
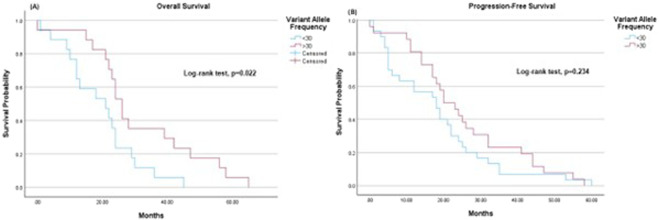
Kaplan-Meier survival estimates. **(A)** Overall survival: The mean overall survival was 19.7 months (95% CI 14.21–25.10) for variant allele frequency <30 and 31.4 months (95% CI 23.15–39.56) for variant allele frequency ≥30. **(B)** Progression-free survival: The mean progression-free survival was 18.2 months (95% CI 12.94–23.46) for variant allele frequency <30 and 25.0 months (95% CI 19.14–30.94) for variant allele frequency ≥30. For patients with EGFR ex19.

When stratified by VAF, patients with VAF <30% had a mean OS of 19.7 months (95% CI: 14.21–25.10), while those with VAF ≥30% had a significantly longer mean OS of 31.4 months (95% CI: 23.15–39.56) (Log-rank test, p = 0.022) (Figure 3A). In terms of PFS, patients with VAF <30% had a mean of 18.2 months (95% CI: 12.94–23.46), while those with VAF ≥30% had a mean PFS of 25.0 months (95% CI: 19.14–30.94), though this difference was not statistically significant (Log-rank test, p = 0.234) (Figure 3B).

**FIGURE 4:**
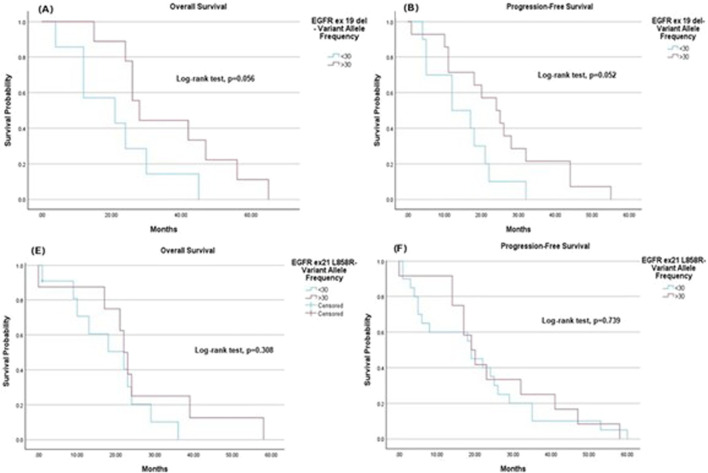
Kaplan-Meier survival estimates. **(A)** Overall survival: the mean overall survival was 21.1 months (95% CI 11.04–31.25) for variant allele frequency <30 and 36.6 months (95% CI 25.61–47.50) for variant allele frequency ≥30. **(B)** Progression-free survival: The mean progression-free survival was 14.8 months (95% CI 9.24–20.37) for variant allele frequency <30 and 24.9 months (95% CI 17.04–32.82) for variant allele frequency ≥30. For patients with EGFR ex21 L858R: **(C)** Overall survival: The mean overall survival was 18.7 months (95% CI 12.36–25.00) for variant allele frequency <30 and 25.5 months (95% CI 13.78–37.22) for variant allele frequency ≥30. **(D)** Progression-free survival: The mean progression-free survival was 19.9 months (95% CI 12.54–27.26) for variant allele frequency <30 and 25.2 months (95% CI 15.92–34.41) for variant allele frequency ≥30.

Among patients with EGFR exon 19 deletion, those with VAF <30% had a mean OS of 21.1 months (95% CI: 11.04–31.25), compared to 36.6 months (95% CI: 25.61–47.50) for VAF ≥30%, indicating a trend toward improved OS (p = 0.056) (Figure 4A). The mean PFS for this group was 14.8 months (95% CI: 9.24–20.37) for VAF <30% and 24.9 months (95% CI: 17.04–32.82) for VAF ≥30% (p = 0.052) (Figure 4B). Similarly, for patients with EGFR exon 21 L858R, the mean OS was 18.7 months (95% CI: 12.36–25.00) for VAF <30% and 25.5 months (95% CI: 13.78–37.22) for VAF ≥30% (p = 0.308), (Figure 4C). The mean PFS was 19.9 months (95% CI: 12.54–27.26) for VAF <30% and 25.2 months (95% CI: 15.92–34.41) for VAF ≥30% (p = 0.739), (Figure 4D).

When comparing outcomes by sex, males demonstrated a trend toward longer survival. The mean OS for females was 22.5 months (95% CI: 16.93–28.11), while for males it was 31.8 months (95% CI: 21.18–42.46) (Log-rank test, p = 0.109). The mean PFS was 19.8 months (95% CI: 14.86–24.73) for females and 25.0 months (95% CI: 18.39–31.60) for males (p = 0.423). Among patients with EGFR exon 19 deletion, the mean OS was 26.0 months (95% CI: 17.10–34.90) for females and 33.6 months (95% CI: 19.49–47.75) for males (p = 0.240). The mean PFS in this group was 17.1 months (95% CI: 12.24–21.90) for females and 25.8 months (95% CI: 15.02–36.58) for males (p = 0.115). For patients with EGFR exon 21 L858R, the mean OS was 21.7 months (95% CI: 13.58–27.87) for females and 27.0 months (95% CI: 14.76–39.24) for males (p = 0.466). The mean PFS was 21.3 months (95% CI: 14.11–28.53) for females and 23.9 months (95% CI: 18.23–29.49) for males (p = 0.998).

Smoking status did not significantly influence the OS and PFS outcomes. The mean OS was 25.4 months (95% CI: 20.28–30.75) for non-smokers and 25.9 months (95% CI: 13.58–38.22) for smokers (Log-rank test, p = 0.731). The mean PFS was 21.4 months (95% CI: 17.38–25.37) for non-smokers and 19.7 months (95% CI: 9.81–29.57) for smokers (p = 0.738). Subgroup analysis of EGFR exon 19 and exon 21 L858R also showed no significant differences in OS or PFS between smokers and non-smokers, with p values were 0.414 and 0.908, respectively.

**FIGURE 5:**
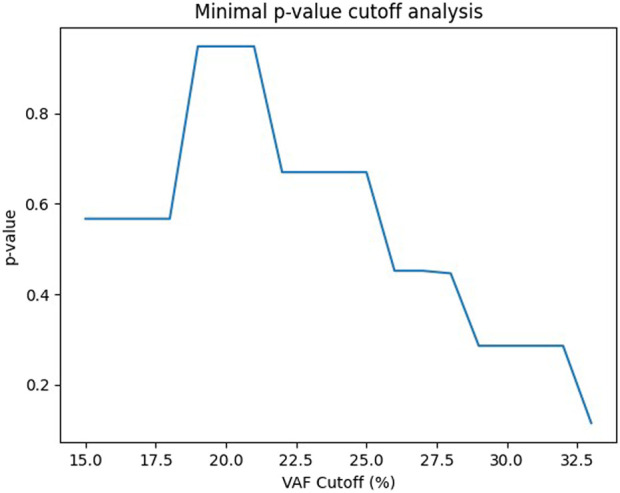
Minimal p-value analysis identifying optimal VAF cutoff.

To determine the optimal VAF threshold, a minimal p-value approach was performed across multiple cut-off values. The analysis identified a VAF threshold of approximately ∼33% as providing the greatest separation in overall survival. This value closely approximates the pre-specified 30% cut-off used in our primary analysis, supporting the statistical validity and robustness of the selected threshold (Figure 5).

Among treatment related adverse events (AEs), in our study, fatigue was the most commonly reported AE, with 27.21% of patients experiencing it as a grade 1 event, followed by 13.61% who experienced it at Grade 2 severity, and 0.68% at grade 3/4 severity. Diarrhea also emerged as a frequently occurring AE, affecting 12.24% of patients at grade 1, 10.20% at grade 2, and 0.68% at grade 3/4. Similarly, rash was commonly observed, with 11.56% of patients reporting it at grade 1, 8.84% at grade 2, and 0.68% at grade 3/4. Other commonly reported grade 1 AEs included neutropenia and stomatitis, both seen in 6.12% of patients, while paronychia and anemia were observed in 5.44% and 4.76% of participants, respectively. In terms of grade 2 AEs, fatigue remained the most prevalent, followed by diarrhea and rash, while neutropenia, thrombocytopenia, and anemia were also frequently reported at this level. Although Grade 3/4 AEs were less common, the most notable was lowering of left ventricular ejection fraction, which was observed in 3.40% of patients, followed by pancytopenia, cerebrovascular accident, leukopenia, atrial fibrillation, and pneumonitis, each reported in 0.68% of participants (Table 2).

**TABLE 2:** Treatment-related adverse events of the Study Population (n = 147).

Adverse event	Grade 1 (frequency, %)	Grade 2 (frequency, %)	Grade 3 and 4 (frequency, %)
Fatigue	40 (27.21)	20 (13.61)	1 (0.68)
Diarrhea	18 (12.24)	15 (10.20)	1 (0.68)
Rash	17 (11.56)	13 (8.84)	1 (0.68)
Neutropenia	9 (6.12)	6 (4.08)	1 (0.68)
Stomatitis	9 (6.12)	4 (2.72)	-
Paronychia	8 (5.44)	7 (4.76)	1 (0.68)
Anemia	7 (4.76)	6 (4.08)	2 (0.68)
Elevated creatinine	6 (4.08)	3 (2.04)	-
Abdominal pain	6 (4.08)	-	-
Thrombocytopenia	6 (4.08)	6 (4.08)	1 (0.68)
Pruritus	4 (2.72)	-	-
Decreased appetite	4 (2.72)	-	-
Vertigo	4 (2.72)	-	-
Lower ejection fraction	-	-	5 (3.40)
Nausea	3 (2.04)	-	-
Pancreatitis	3 (2.04)	-	-
Pancytopenia	2 (1.36)	-	1 (0.68%)
Back pain	2 (1.3%)	-	-
Alopecia	1 (0.68)	3 (2.04)	-
Headache	2 (0.68)	1 (0.68)	-
Constipation	3 (0.68)	-	-
Hyponatremia	4 (0.68)	-	-
Hypercalcemia	5 (0.68)	-	-
Deep vein thrombosis	6 (0.68)	-	-
Peripheral neuropathy	-	1 (0.68)	-
Cerebrovascular accident	-	-	1 (0.68)
Leukopenia	-	-	1 (0.68)
Atrial fibrillation	-	-	1 (0.68)
Pneumonitis	-	-	1 (0.68)

To provide comparative context for the prognostic value of EGFR VAF, we compared our findings with previously reported studies evaluating VAF in EGFR-mutant NSCLC treated with earlier-generation TKIs. Prior studies evaluating EGFR VAF in patients treated with first- and second-generation TKIs reported mixed results, with some demonstrating improved progression-free survival among patients with higher VAF, while others showed no significant association with survival outcomes. In our cohort of patients treated uniformly with first-line osimertinib, higher VAF (≥30%) was associated with significantly improved overall survival and a trend toward improved progression-free survival. These findings suggest that VAF may retain prognostic significance in the modern osimertinib treatment era.

## 4 Discussion

In the era of precision and personalized cancer therapy, accurately defining tumor type—including comprehensive histological classification and detailed molecular pathological features—is essential. Targeting EGFR represents a promising approach for treating lung adenocarcinoma patients, as numerous studies over the past decade have demonstrated the effectiveness of TKIs like osimertinib for advanced and locally staged NSCLC with EGFR sensitizing mutations. However, the efficacy of TKIs varies among patients, and not all individuals with EGFR-activating mutations experience similar OS and PFS (Zhang et al., 2016; Tan et al., 2015; Tan et al., 2018).

The allelic fraction is affected by factors such as the proportion of tumor cells in the sample, the presence of copy number alterations, and, most importantly, the percentage of cells within the tumor that carry the mutation. Mutations that arise early in tumor development, known as clonal or truncal mutations, typically have a higher allelic frequency than those that occur later as subclonal mutations. These early mutations are more commonly drivers of cancer progression and represent promising therapeutic targets (Fri et al., 2021; Gieszer et al., 2021; Li et al., 2019).

Currently, the biological and clinical significance of VAF for EGFR mutations in NSCLC patients, particularly regarding prognosis and response to osimertinib, remains largely unclear. Additionally, there is limited literature examining the impact of EGFR allelic frequency in NSCLC, and the existing data are inconsistent. From a biological standpoint, it could be inferred that a higher allelic frequency might correlate with a greater likelihood of the variant serving as an oncogenic driver.

In a retrospective analysis of the phase III CTONG 0901 trial, which compared erlotinib and gefitinib in advanced NSCLC with EGFR exon 19 or 21 mutations, the median mutant allelic frequency was 25.8% (range 1.4%–86.2%) among 105 patients. Patients were divided into two groups: high (25.8%–86.2%) and low (1.4%–25.8%) allelic frequency. The analysis found no significant differences between the groups in overall response rate (56.2% vs. 57.5%), progression-free survival (11.2 vs. 12.4 months), or overall survival (20.5 vs. 23.1 months), with a P-value of 0.500 (Li et al., 2019).

Another study indicated that the mutant allelic frequency of EGFR in NSCLC may be linked to clinical outcomes in patients treated with TKIs. Although the study had a small cohort of 42 patients who received various first-line TKIs (gefitinib, erlotinib, afatinib, osimertinib, dacomitinib) and used a simple cut-off of 0.30 to separate low from high allelic frequency, the researchers observed a clear improvement in progression-free survival (PFS) for patients with a high EGFR allelic frequency compared to those with a low frequency. However, overall survival (OS) differences were not significant (Fri et al., 2021).

A real-world study involving 89 patients assessed the efficacy of EGFR VAF and found a positive linear correlation between EGFR-VAF in tumoral tissue and progression-free survival (PFS) (r = 0.319; P = 0.002). High EGFR-VAF (≥70%) was identified as an independent predictor of longer PFS compared to low EGFR-VAF (<70%), with median PFS of 52 weeks *versus* 26 weeks, respectively (P < 0.001). Additionally, patients with high EGFR-VAF demonstrated significantly improved overall survival (OS) compared to those with low EGFR-VAF (P = 0.011). However, this study included patients who were treated with either gefitinib or erlotinib as first- or second-line therapies (Gieszer et al., 2021).

Since the FLAURA and FLAURA two trials (Leighl et al., 2020; Ramalingam et al., 2020; Planchard et al., 2023) have established osimertinib as the current standard of care for patients with EGFR-mutant NSCLC, the previously mentioned studies, which involved first-line treatments like gefitinib, erlotinib, afatinib, and dacomitinib, are now less relevant (Fri et al., 2021; Gieszer et al., 2021; Li et al., 2019; Leighl et al., 2020; Ramalingam et al., 2020; Planchard et al., 2023). In contrast, our study specifically examined the current first-line standard of care, osimertinib, and provides the largest real-world data available—addressing a key limitation of earlier studies.

Furthermore, in our cohort of EGFR-mutant NSCLC patients treated with osimertinib, we found that VAF was associated with improved PFS and OS across the overall population and within subgroups such as EGFR exon 19 deletions and exon 21 L858R mutations. Our findings suggest that EGFR VAF may represent a prognostic biomarker in EGFR-mutant NSCLC patients treated with osimertinib. However, because all patients in our study received osimertinib, it remains unclear whether VAF is predictive of response to osimertinib specifically or reflects a broader biological phenomenon related to tumor clonality and heterogeneity.

Using a minimal p-value approach, we identified an optimal VAF threshold of approximately 33%, which closely aligns with the 30% cut-off used in our primary analysis. This finding supports the biological and clinical rationale for selecting this threshold. While VAF represents a continuous variable, the use of a clinically meaningful cut-off may facilitate risk stratification and clinical decision-making in EGFR-mutant NSCLC.

Importantly, because our cohort included only patients treated with osimertinib, we cannot determine whether VAF represents a predictive biomarker specific to osimertinib response or a general prognostic biomarker in EGFR-mutant NSCLC. The association between higher VAF and improved outcomes may reflect tumor clonality, with higher VAF indicating truncal driver mutations and lower tumor heterogeneity, which has been associated with improved treatment outcomes across multiple therapies. Future studies comparing different treatment modalities or including control cohorts will be required to clarify this distinction.

Several studies have investigated the relationship between EGFR VAF and clinical outcomes in EGFR-mutant NSCLC, supporting the hypothesis that VAF reflects tumor clonality and biological aggressiveness. Additionally, a study by Anna Buder and colleagues specifically evaluated EGFR mutation allele frequency in patients treated with osimertinib after progression on earlier EGFR-TKIs and found that higher allele frequency was associated with improved survival outcomes, supporting a potential prognostic role of VAF even in the context of third-generation EGFR inhibitors (Buder et al., 2021). From a biological perspective, tumor heterogeneity has been increasingly recognized as a key determinant of resistance to targeted therapies. Single-cell analysis studies demonstrated that EGFR-mutant NSCLC tumors exhibit substantial intratumoral heterogeneity, and patients with a higher proportion of EGFR-mutant cells showed longer progression-free survival compared to those with more heterogeneous tumor populations. These findings support the hypothesis that higher VAF may reflect dominant clonal driver mutations and reduced heterogeneity, leading to improved outcomes across targeted therapies rather than a treatment-specific effect limited to Osimertinib (Guo et al., 2019). Furthermore, a comprehensive review highlighted that variant allele frequency is influenced by tumor cellularity, copy number alterations, and clonal architecture, and may serve as a practical surrogate marker of tumor clonality. The authors hypothesized that tumors with lower allelic frequency may respond less favorably to targeted therapies due to subclonal mutation distribution and increased tumor heterogeneity (Boscolo et al., 2023).

A limitation of our study is its retrospective nature and the absence of randomization. Additionally, there is no well-defined cut-off for allelic frequency to classify EGFR mutations, and the method for determining this threshold is not standardized. We based our cut-off on the visual assessment of tumor cellularity by an experienced molecular pathologist, assuming that a homogeneous population of EGFR-mutant cells would yield an allelic frequency representing at least half of the tumor cellularity, corresponding to one mutant allele for every two alleles (the other being normal). While this assumption is generally applicable, it may not always hold due to factors such as the amplification of the normal allele, though no selective pressure favors this allele. It is also uncertain whether this cut-off will apply to other cohorts, but our approach is rooted in general principles rather than being specific to our institution or the time frame of data collection.

Finally, the key questions that emerge from our findings are whether adding chemotherapy to osimertinib for patients with a VAF less than 30 could improve outcomes, and whether combining chemotherapy with osimertinib for patients with a VAF above 30 might further enhance their prognosis. These considerations could potentially guide more personalized treatment strategies for EGFR-mutant NSCLC, depending on VAF levels.

## 5 Conclusion

Our study found that the VAF is a key predictor of OS and PFS in EGFR mutant patients treated with osimertinib. Patients with a VAF of 30% or higher had significantly better OS and PFS compared to those with a VAF below 30%. Specifically, those with higher VAF experienced longer mean OS across the overall cohort as well as in subgroups with EGFR exon 19 deletions and exon 21 L858R mutations. These results suggest that VAF could be an important predictive biomarker for treatment outcomes in EGFR-mutated NSCLC patients, underscoring its potential to help tailor treatment strategies.

## Data Availability

The raw data supporting the conclusions of this article will be made available by the authors, without undue reservation.
